# MicroRNA panels as disease biomarkers distinguishing hepatitis B virus infection caused hepatitis and liver cirrhosis

**DOI:** 10.1038/srep15026

**Published:** 2015-10-12

**Authors:** Bo-Xun Jin, Yong-Hong Zhang, Wen-Jing Jin, Xiang-Ying Sun, Gui-Fang Qiao, Ying-Ying Wei, Li-Bo Sun, Wei-Hong Zhang, Ning Li

**Affiliations:** 1 Beijing YouAn Hospital, Capital Medical University, No.8, Xitoutiao, Youanmenwai, Fengtai District, Beijing, China; 2 Beijing QuantoBio Biotechnology Co. Ltd, No.1, Disheng East Road, Beijing Economic-Technological Development Area, Daxing District, Beijing, China

## Abstract

An important unresolved clinical issue is to distinguish hepatitis B virus (HBV) infection caused chronic hepatitis and their corresponding liver cirrhosis (LC). Recent research suggests that circulating microRNAs are useful biomarkers for a wide array of diseases. We analyzed microRNA profiles in the plasmas of a total of 495 chronic hepatitis B (CHB) patients, LC patients and healthy donors and identified 10 miRNAs that were differentially expressed between CHB and LC patients. Our logistic models show that three panels of miRNAs have promising diagnostic performances in discriminating CHB from LC. Blinded tests were subsequently conducted to evaluate the diagnostic performances in clinical practice and a sensitivity of 85% and specificity of 70% have been achieved in separating CHB from LC pateints. The expression levels of some circulating miRNAs were significantly correlated with HBV DNA load and liver function, such as prothrombin activity (PTA) and levels of alanin aminotransferase (ALT), albumin (ALB) and cholinesterase (CHE). Our results provide important information for developing novel diagnostic tools for distinguishing chronic HBV hepatitis and their corresponding cirrhosis.

Chronic HBV infection is an important cause of viral hepatitis (CHB) and liver cirrhosis (LC) worldwide. It is estimated that there are more than 350 million people with chronic HBV infection and more than one million patients die each year^1^. Despite effective vaccination that has resulted in a decrease of acute HBV incidences in many countries, persistent infection of HBV remains a principle challenge. Therefore, it is very important to have accurate method to diagnose and evaluate CHB and LC related to chronic HBV infection (LC for short in the following part) safely and conveniently.

HBV infection and its disease progression are often monitored by clinical parameters. Biopsy is an invasive method with good diagnostic performance, but for its risk of various complications, it is difficult to be performed routinely. Noninvasive methods include blood tests and image examination, including aminotransferases (ALT, AST), fibrosis related factors, HBV DNA levels and the presence of HBV antigens^2^,^3^,^4^, but these clinical parameters have limited use in distinguishing patients who have chronic hepatitis from those who have developed cirrhosis^5^. None of the methods could discriminate CHB patients from chronic HBV carriers with no clinical symptoms or discriminate LC patients at the early stage from CHB patients. Based on the reasons above, it is urgently needed to develop noninvasive predictive biomarkers and methods to improve the diagnostic accuracy for CHB and LC.

miRNAs are a class of small noncoding RNAs with ~22 nucleotides that regulates 20–80% of the host genes^6^. Previous studies show that multiple miRNAs are involved in liver metabolism, liver fibrosis and regeneration, as well as HCC^7^,^8^,^9^. Studies also demonstrate that miRNAs in serum or plasma could discriminate HCC from other liver diseases^10^. Early diagnosis and effective treatment of cirrhosis would reduce the possibilities of progression to HCC^11^. Yan-Jie Chen *et al.* found that a combination of the circulating miR-106b and miR-181b in plasma may be used for liver cirrhosis diagnosis with an AUC of 0.7 ~ 0.8^12^. However, miRNA expression profiles within different stages of CHB and LC are not well defined.

In this study, we analyzed the expression profiles of 440 miRNAs in the plasmas of CHB, LC patients and healthy subjects using real-time PCR. 10 candidate miRNAs were identified and further validated in an independent training and validation cohort. Based on the differential expression data, we have established logistic models with promising diagnostic performances. Our findings provide useful information for developing novel tools for the diagnosis of HBV infection induced chronic hepatitis and cirrhosis.

## Results

### Profiling of circulating miRNAs in chronic HBV infection caused hepatitis and liver cirrhosis

**Figure 1:**
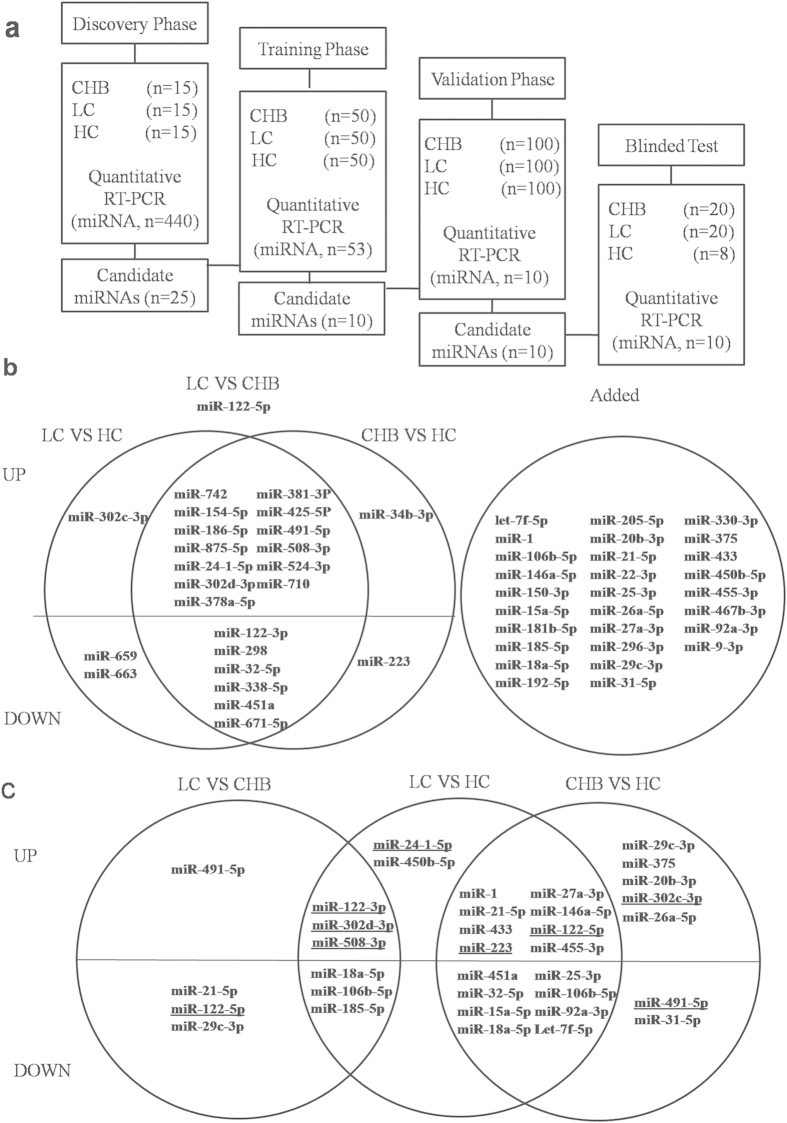
Profiling of miRNAs. (**a**) Study design, Chronic hepatitis B (CHB) patients, liver cirrhosis (LC) patients and healthy controls (HC) were recruited for the discovery (15/group), training (50/group), validation (100/group) phases, and the blinded test. Individual miRNAs in the plasma were measured using quantitative realtime-PCR assay. (**b**) Profiling of miRNAs in training phase of HBV infected patients. (**c**) Profiling of miRNAs in validation phase of HBV infected patients. MiRNAs with significantly different expression levels between CHB, LC and HC of training phase (**b**) and validation phase (**c**) were shown. MiRNAs up indicates up-regulation while down meant down-regulation. Lined showed same miRNAs had different expression levels in different groups.

To search for circulating miRNA biomarkers for chronic HBV infection induced hepatitis and cirrhosis, we have recruited a total of 495 CHB, LC and healthy controls (HC) for the discovery, training and validation phases of a typical biomarker discovery process (Fig. 1a, Table 1). In addition, we have recruited a total of 40 patients and 8 HC for the blinded test.

Circulating miRNAs consisting of 440 individual miRNAs in the plasmas were measured using real time-PCR assay. In the discovery phase, the expression of 25 miRNAs was found to be differentially expressed between CHB, LC or HC groups (*p* < 0.05, Fig. 1b), 13 of them were up-regulated and 6 of them were down-regulated in both CHB and LC group than in HC group.

**Figure 2:**
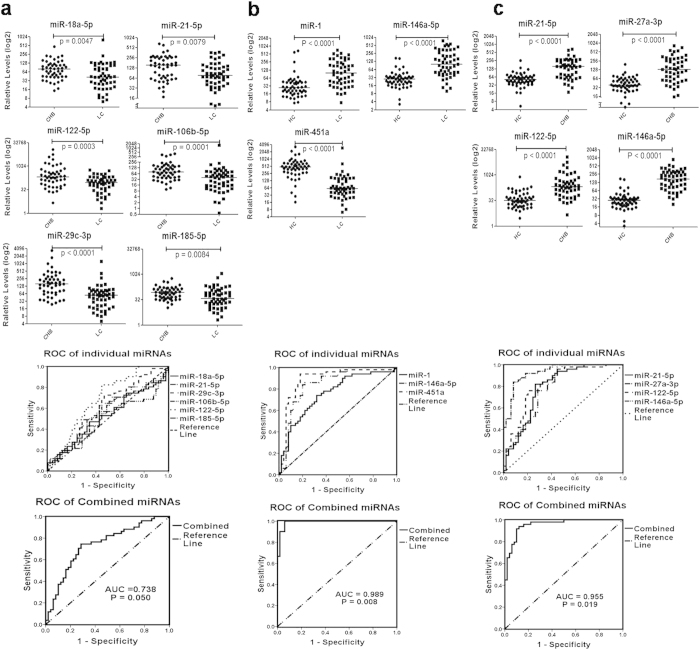
Expression of plasma miRNAs among study cohorts in training phase. Relative expression levels of the miRNAs in plasma and ROC analysis with individual miRNAs and combined miRNA panel between LC and CHB patients (**a**), LC patients and HC (**b**), and CHB patients and HC (**c**) in training phase. P values were calculated using the Mann-Whitney test and p were all <0.01. Logistic regression indicates a linear combination of miRNAs.

The expression profiles of the 25 above-identified miRNAs and 28 more miRNAs reported in other studies on CHB or LC^13^,^14^,^15^,^16^,^17^,^18^,^19^,^20^,^21^,^22^,^23^ were further tested in an independent training set. Among the 53 miRNAs, 33 miRNAs were differentially expressed among HC, CHB and LC subjects (Fig. 1c). Ten of the miRNAs discriminated CHB or LC from HC group (6 miRNAs for LC versus CHB, 3 miRNAs for LC versus HC and 4 miRNAs for CHB versus HC, Table 2). Paired-comparison of the relative expression levels of the 6 miRNAs revealed that all the miRNAs (miR-18a-5p, miR-21-5p, miR-29c-3p, miR-106b-5p, miR-122-5p and miR-185-5p) were down-regulated in LC patients when compared with those in CHB patients (Fig. 2a). ROC analysis was performed to evaluate the diagnostic performances of the miRNAs to discriminate these groups. None of the miRNAs had an AUC value greater than 0.8 between CHB and LC group and combined miRNA panel showed an AUC value of 0.738 (Fig. 2a).

Most of the miRNAs were up-regulated in LC (miR-1 and miR-146a-5p, Fig. 2b) or CHB (miR-21-5p, miR-27a-3p, miR-122-5p and miR-146a-5p, Fig. 2c) patients when compared with HC controls. Only miR-451a had lower expression in LC patients than that in HC controls (Fig. 2b). ROC analysis demonstrates that majority of these miRNAs (6/7, 85.7%) had an AUC value >0.8 when their expression in LC (Table 2 and Fig. 2b) or CHB (Table 2, Fig. 2c) patients were compared to those in HC controls. These findings indicate that the identified miRNAs could efficiently discriminate between CHB/LC patients and HC subjects. The AUCs of the 2 panels were 0.955 for CHB and 0.989 for LC.

### Validation of miRNAs for diagnosis of HBV infection induced chronic hepatitis and liver cirrhosis

**Figure 3:**
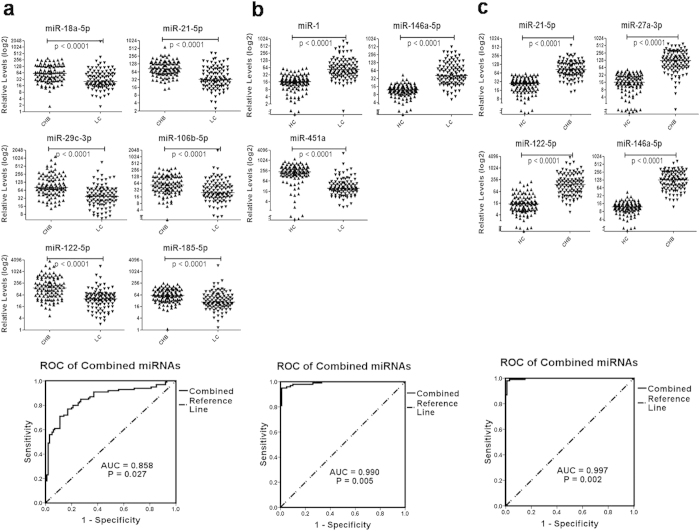
Expression of miRNAs among study subjects in the validation phase. Relative expression levels of the miRNAs in plasma and ROC analysis with combined miRNA panel between LC and CHB patients (**a**), LC patients and HC (**b**), and CHB patients and HC (**c**) in validation phase. P values were calculated using the Mann-Whitney test and p were all <0.01. Logistic regression indicates a linear combination of miRNAs.

The expression profiles of the 10 miRNAs were further validated in an independent cohort including 100 HC, 100 CHB and 100 LC subjects. The expression patterns of all the 10 miRNAs among the three groups were consistent with those in the training phase although the significance of the differences with each miRNA from paired comparison slightly varied (Fig. 3). ROC analyses of the diagnostic performances of the miRNA panels show that the AUC value of the miRNA panel (miR-18a-5p, miR-21-5p, miR-29c-3p, miR-106b-5p, miR-122-5p and miR-185-5p) for the diagnosis of CHB from LC reached 0.858 (95% CI, 0.782-0.895) (Fig. 3a). Moreover, miRNA panels in CHB (miR-21-5p, miR-27a-3p, miR-122-5p and miR-146a-5p) or LC (miR-1, miR-146a-5p and miR-451a) patients could readily distinguish from the HC controls with AUC values close to 1.0 (0.990 for LC versus HC and 0.997 for CHB versus HC, Fig. 3b,c). With logistic regression analysis of validation datasets, the miRNAs that were differentially expressed in HBV infection induced CHB and LC were combined to build three models for the diagnosis of HBV infection induced liver diseases (CHB and LC) from HC subjects and the discrimination of CHB and LC patients (Table 3). The cut-off values of the diagnostic performances of the models were determined based on the maximum of Youden index of the ROC curve (P > 0.6027 for LC versus CHB, P > 0.3041 for LC versus HC, P > 0.4325 for CHB versus HC). Greater than 95% specificity and sensitivity were demonstrated between CHB or LC groups and the HC subjects while a sensitivity of 80% and a specificity of 70% were obtained for the diagnosis of LC from CHB patients.

### Blinded evaluation of the diagnostic performances of the miRNA panels

We then performed a double-blinded test on 48 subjects to verify the diagnostic performances of the miRNA panels in clinical practice. The researchers were blinded to the diagnostic information while the doctors responsible for sample collection were blinded to the test results. The sensitivity and specificity were calculated based on the models established above. The results showed that all of the CHB and LC patients could be discriminated from HC controls with both sensitivity and specificity of 100%. For CHB and LC patients, results based on the miRNA models reached a sensitivity of 85% and a specificity of 70%.

### Correlations of miRNAs with liver synthesis and metabolism ability in LC patients

**Figure 4:**
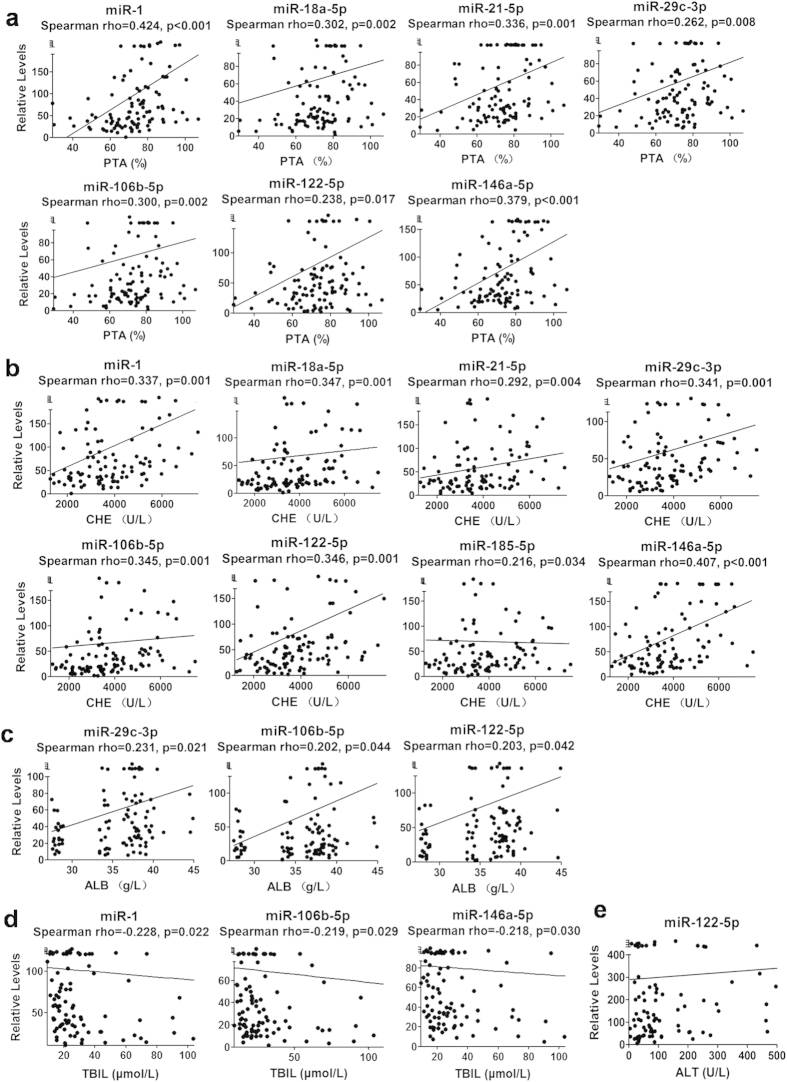
Correlations of miRNAs expression and liver function in patients of validation phase. Correlations of miRNAs expression and PTA (**a**), CHE (b), ALB (**c**) and TBIL(**d**) levels in LC patients, and correlation of miRNA expression and ALT (**e**) levels in CHB patients. Y axis represents relative expression levels of each miRNA in the corresponding subjects. *P* values were calculated using the Mann-Whitney test and *p* < 0.05 was significant.

Pro-thrombin activity (PTA), the levels of serum albumin (ALB) and cholinesterase (CHE) are indicators of hepatic synthetic function. Correlations between the differentially expressed miRNAs and these indicators were analyzed in LC patients of the validation phase. Seven miRNAs (miR-1, miR-18a-5p, miR-21-5p, miR-29c-3p, miR-106b-5p, miR-122-5p and miR-146a-5p, Fig. 4a) had significant positive correlations with PTA (p < 0.05) while 8 miRNAs (miR-1, miR-18a-5p, miR-21-5p, miR-29c-3p, miR-106b-5p, miR-122-5p, miR-185-5p and miR-146a-5p, Fig. 4b) had significant positive correlations with the level of CHE (p < 0.05 ). Three miRNAs (miR-29c-3p, miR-106b-5p and miR-122-5p, Fig. 4c) had significant positive correlations with the level of ALB (p < 0.05). The levels of serum total bilirubin (TBIL) are an indicator of hepatic metabolism ability. Three miRNAs (miR-1, miR-106b-5p, miR-146a-5p, Fig. 4d) had significant negative correlations with TBIL (p < 0.05).

### Correlations of miRNAs with ALT levels in CHB patients

We also analyzed the correlation of miRNA levels and the serum level of ALT in CHB patients in the validation phase as ALT is most widely used indicator of liver injury. The results show that only miR-122-5p had a significant positive correlation with ALT while the other 3 miRNAs had no significant correlations (Fig. 4e).

### Expression profiles of the miRNAs in different states of LC patients

**Figure 5:**
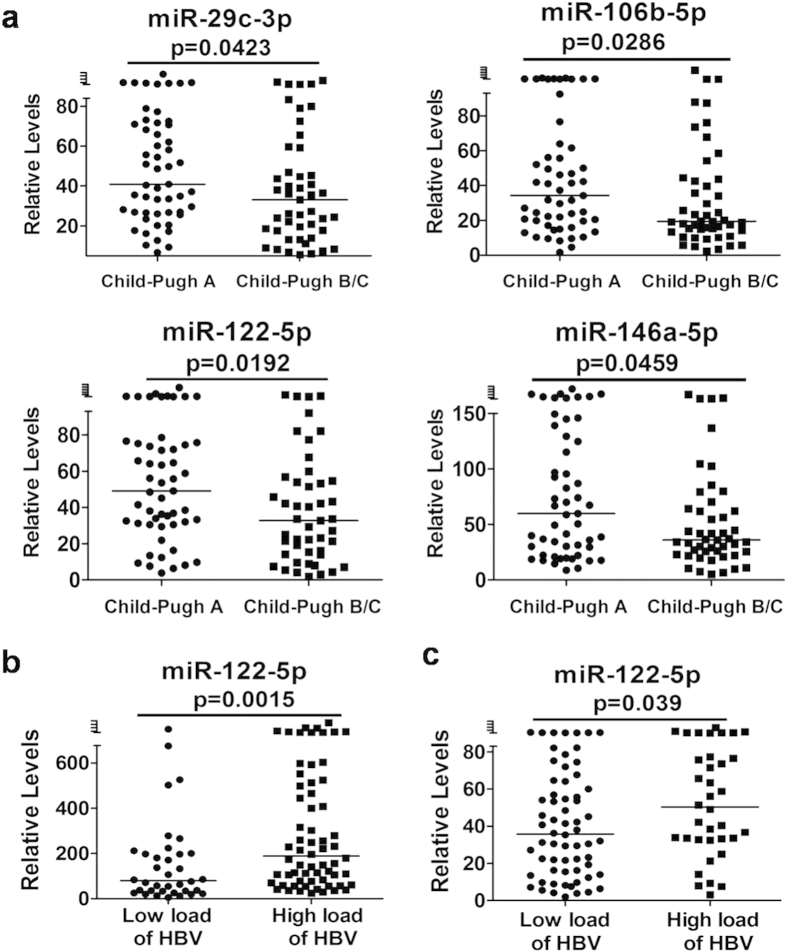
Comparison of miRNAs in different status of CHB and LC patients. (**a**) 4 miRNAs, miR-29c-3p, miR-106b-5p, miR-122-5p and miR-146a-5p were significantly upregulated in Child-Pugh A LC patients (p < 0.05) compared with Child-Pugh B/C LC patients. (**b,c**) According to serum levels of HBV load, CHB patients and LC patients were separated into 2 groups, miR-122-5p was considerably up-regulated in CHB (p = 0.0015, b) or LC (p = 0.039, c) patients with high viral load than with low viral load. Y axis represents relative expression levels of each miRNA in the corresponding subjects. P values were calculated using the Mann-Whitney test and p < 0.05 was significant.

Taking consideration of their synthetic and metabolism ability, liver function of LC patients can be characterized into three states (Grade A, B and C) according to Child-Pugh score. We separated the LC patients in the validation phase into two categories (53 patients with liver function in Grade A and 47 patients with liver function in Grade B or C). Four miRNAs, miR-29c-3p, miR-106b-5p, miR-122-5p and miR-146a-5p, were significantly up-regulated in patients in Grade A (p < 0.05) compared with patients in Grade B/C, while other miRNAs showed no significant differences between the two categories (Fig. 5a). However, logistic regression and ROC analysis of these 4 miRNAs show that they could not discriminate the two groups (data not show).

### Expression profiles of the miRNAs in different viral status of CHB and LC

We next sought to find out the expression profiles of miRNAs in different viral status of CHB and LC. According to serum levels of HBV load in CHB and LC patients, we took 2 × 10^4^ U/ml as the cut-off value and separated CHB patients and LC patients into 2 groups: patients with high virus load and patients with low virus load^24^,^25^. In the 100 CHB patients in the validation phase, 64 patients had high virus load and 36 patients had low virus load. And in the 100 LC patients in the validation phase, 36 patients had high virus load and 64 patients had low virus load. Interestingly, miR-122-5p was significantly up-regulated in CHB (p = 0.0015, Fig. 5b) or LC (p = 0.039, Fig. 5c) patients with high load of HBV than with low load.

## Discussion

Circulating miRNAs as diagnostic markers for different cancers were extensively studied in recent years. In HBV driven HCC, miRNAs has been found to played critical functional roles. During different status of CHB and LC, the profiling of miRNA is also important for understanding the mechanisms of HBV driven diseases progression. MiR-106b and miR-181b may be biomarkers for liver cirrhosis in plasma^12^. MiR-885-5p was up-regulated in serum of HBV, LC and HCC patients, and could be a potential marker for liver pathologies^26^. Let-7c, miR-23b, miR-122, miR-150 and miR-122-5p, miR-192-5p were found separately to be novel signatures for hepatitis B virus infection^27^,^28^. Serum miR-143 and miR-215 were potential biomarkers for CHB and hepatocellular carcinoma^29^. However, miRNAs for distinguishing liver cirrhosis from chronic HBV hepatitis, had not been well studied.

In this study, we identified 10 individual miRNAs with distinct expression profiles between HC, CHB and LC. Through the determination and validation in independent training phase, validation phase and blinded test, 3 logistic models with different miRNA panels were established to discriminate CHB and LC from healthy subjects. Of 10 miRNAs that were differentially expressed in CHB and LC, one was for only CHB detection (miR-27a-3p), six were for only LC detection (miR-1, miR-451a, miR-18a-5p, miR-29c-3p, miR-106b-5p and miR-185-5p) and three were for both CHB and LC detection (miR-21-5p, miR-122-5p and miR-146a).

Measuring the degree of liver injury caused by infection and inflammation is the main tool to diagnose CHB, and the circulating level of ALT is the primary indicator clinically. However, several studies have shown that in CHB patients, the level of ALT did not correlate with the pathological result well^30^ In some cases, G2 or higher degrees of liver inflammation were detected through liver biopsy while the serum level of ALT remained normal^31^. This phenomenon may be explained by the slow change of ALT. We evaluated the correlations between ALT level and four miRNAs for CHB, which were considered to change more rapidly than traditional biochemical markers^32^. There were no significant correlations between the levels of three miRNAs (miR-21-5p, miR-27a-3p and miR-146a-5p) and ALT. The level of miR-122-5p was correlated with the level of ALT, but the correlation is low (r = 0.2755, p = 0.0058). Therefore, we consider that the four miRNAs were independent and could supplement with the level of ALT for diagnosis. Some studies show correlations between circulating levels of miRNAs and degrees of liver inflammation through pathological assay^33^,^34^. Future investigation is needed to determine the correlation between the four miRNAs and liver pathology to see whether the levels of miRNAs could reflect the degrees of liver inflammation.

LC patients could develop hepatic decompensation and related complications, such as liver failure. It is very important to evaluate the compensatory capability of liver function through the observation of hepatic synthetic function (synthesis of ALB, CHE and coagulation factors) and metabolic function (metabolism of bilirubin). To evaluate the relationship between the miRNA markers for LC and the compensatory capability of liver function, we examined the correlations between the levels of the miRNAs and PTA, and the levels of ALB, CHE and TBil. We found that 8 miRNAs were correlated with hepatic synthesis function positively and 3 miRNAs were correlated with hepatic metabolic function negatively. We further used the Child-Pugh score to categorize the LC patients into two groups, Child-Pugh A and Child-Pugh B/C. We found that the 4 miRNAs were down-regulated as deterioration of liver compensatory ability and had significant differences between the two groups. Our results indicate that these miRNAs can be used to evaluate the degree of liver decompensation in addition to diagnose of LC.

We evaluated the correlation between viral load and the levels of the eleven miRNAs. We found that only miR-122-5p expression is significantly different between patients with high load and low load of virus while the expression levels of the other miRNAs show no differences between the two groups. This result indicates that the expression of these miRNAs was not affected by HBV viral load. These miRNAs were more likely related to liver inflammation and liver fibrosis. Although we found significant correlation between the level of miR-122-5p and HBV virus load, miR-122-5p may be a marker for liver dysfunction as several studies report that miR-122-5p is up- or down-regulated in liver diseases by other causes^17^,^35^ and the level of this miRNA was also correlated with HCV viral load in CHC patients^36^.

The identified miRNAs have diverse biological functions. To date, the liver specific miR-122 was extensively studied in liver associated diseases^37^. miR-122 could negatively regulate the viral gene expression and replication by direct binding to a highly conserved sequence of HBV, and down-regulation of miR-122 in the liver could impact HBV replication and contribute to the persistence of HBV infection and liver damage^9^,^38^. Downregulation of miR-29c induced by TGF-β plays important roles in liver fibrosis and is correlated with HCC progression^39^,^40^. MiR-29c-5p plays a role in development of fibrosis in many organs, including liver, and it is a potential target for fibrosis treatment. miR-18a was a member of miR18-27 cluster which were important in HBV infection and HCC. Up-regulation of miR-106b was observed in hepatocellular carcinoma tumor samples^41^. Further investigations of the roles of miR-29c-3p, miR-106b-5p and miR-18a-5p in the establishment of liver cirrhosis would improve our understanding of the mechanisms of liver damage. MiR-21-5p could serve as a biomarker for viral hepatitis^42^,^43^ and alcoholic liver disease^44^. MiR-27a-3p could serve as a biomarker for chronic hepatitis C^45^. MiR-146a-5p could serve as a biomarker for acute rejection after liver transplantation^46^. However, there are no studies reporting miR-185a-5p, miR-1 and miR-451a as biomarkers for CHB and LC. No miRNA biomarkers have been confirmed as specific to HBV infection in the past and some of miRNAs identified in our study were not reported before as biomarkers for CHB and LC.

In conclusion, we have identified a panel of circulating miRNAs that were deregulated in CHB or LC patients which could efficiently discriminate CHB and LC from healthy subjects. Further analysis of the expression profiles of the miRNAs in different stages of CHB and LC patients revealed that these miRNAs were distinctly expressed in certain stages of the diseases. Some of the miRNAs were significantly correlated with indicators for liver damages. All these findings would benefit our understanding of the expression profiles of circulating miRNAs in different stages of HBV driven diseases and moreover to the development of novel diagnostic tools for the identification of CHB and LC.

## Methods

### Participants

495 venous blood samples with CHB (n = 165), liver cirrhosis (n = 165) and healthy subjects (n = 165) were collected from Beijing You-An Hospital between July 2009 and June 2013. Additional cohorts of 48 subjects were also recruited to this study for blinded test. All the patients were diagnosed based on clinical and laboratory results. Specificlly, CHB patients had serum positive for HBsAg and HBV DNA longer than six months; had a history of impaired liver function; had no presence of portal hypertension. LC patients had a history of HBsAg and HBV DNA to be positive longer than six months; had no presence of portal hypertension; had no esophageal gastric varices, which is confirmed by enhance CT scan and gastroscopy. Patients with chronic liver disease attributed to causes other than HBV infection were excluded. Written informed consent was obtained from each of the participants and the study was approved by the Ethics Review Committee (ERC) of Beijing You-An Hospital. The study was conducted in accordance with the principles of the Declaration of Helsinki, the standards of Good Clinical Practice (as defined by the International Conference on Harmonization).

### Study procedures

Step-by-step procedures including the discovery phase, the training phase, the validation phase, and the blinded test phase were designed and carried out as listed in Fig. 1a. In the discovery phase, plasmas from 15 CHB, 15 LC patients and 15 healthy subjects were collected. The expression profiles of 440 miRNAs were determined among the three groups and candidate miRNAs with differential expression (Mann–Whitney U test, *p* < 0.05) were identified for further investigation. In the training phase, the 53 identified miRNAs were tested in the plasma samples from independent cohorts consisting of 50 CHB, 50 LC and 50 healthy subjects. 10 miRNAs which were differentially expressed (Mann–Whitney U test, p < 0.05) among the three subject groups were further validated in the next phase. In the validation phase, the 10 identified miRNAs were further validated in an independent large cohort of 300 subjects including CHB, LC and healthy subjects. In the blinded test phase, the clinical diagnostic performances of the miRNA panels were tested in which the researchers were blinded to the clinical diagnostic information and the physicians were blinded to the test results. After unblinding the clinical diagnostic information, sensitivity and specificity of the logistic models in discriminating CHB, LC and healthy subjects were calculated.

### Plasma miRNA quantification

Vein blood samples were collected by BD Vacutainer Plus and centrifuged at 3000 rpm for 15 min within 4 h. The supernatant plasmas were immediately recovered and stored at −80 ^o^C. Plasma miRNAs were extracted using Total RNA Isolation Kit (QuantoBio). Synthetic Caenorhabditis elegans miRNA (cel-miR-67) was used as a exogenous control and added to the plasma lysate before extraction. Quantities of miRNAs were determined by SYBR-based qRT-PCR according to manufactures’ instructions (Quantobio). Briefly, Escherichia coli polyA polymerase was used to add a polyA tail at the 3′ end of RNA molecules. With oligo(dT) annealing, a universal tag was attached to the 3′ end of cDNAs during the cDNA synthesis using reverse transcriptase (Quantobio). Quantitative PCR was performed with miRNA-specific forward primers and a universal reverse primer mix according to the universal tag.

### Data analysis

Plasma miRNA levels among different groups of subjects were normalized by exogenous control: cel-miR-67, and analyzed with RealTime StatMiner (Integromics) and miRNAs with differential expression (Mann–Whitney U test, p < 0.05) were sorted out for further analysis. Logistic regression indicates a linear combination of miRNAs. Receiver-operating characteristic (ROC) curves and the area under the ROC curve (AUC) were used to assess the sensitivity and specificity of miRNA biomarkers and construct the diagnostic models based on the predicted probability. The relationship between the miRNAs and liver function was visualized by Spearman rank correlation analysis, and *p* < 0.05 (Mann-Whitney test) indicated statistical significance. Statistical evaluation was performed using the Statistical Package for the Social Sciences (SPSS 13.0) and graphs were generated using GraphPad Prism 5.0 (GraphPad Software).

## Additional Information

**How to cite this article**: Jin, B.-X. *et al.* MicroRNA panels as disease biomarkers distinguishing hepatitis B virus infection caused hepatitis and liver cirrhosis. *Sci. Rep.* **5**, 15026; doi: 10.1038/srep15026 (2015).

## Figures and Tables

**Table 1:** Characteristics of Participants in Study.

Group	Characteristic	Discovery Phase	Training Phase	Validation Phase	Blinded Test	*p* value^d^
CHB^a^	Number	15	50	100	20	
	Age (mean,SD)	43, 16	40, 14	41, 14	43, 17	0.19
	Gender (male/Female)	10/5	34/16	71/29	13/7	0.95
	ALT (U/L,median,range)	—	82, 22-962	54, 5–2143	53, 29–487	0.79
	HBV-DNA (log10 IU/mL,mean,SD)	—	5, 2	5, 2	3, 2	0.57
LC^b^	Number	15	50	100	20	
	Age (Mean,SD)	44, 14	45, 12	43, 10	47, 17	0.74
	Gender (male/Female)	10/5	36/14	68/32	13/7	0.94
	Severity of LC (compensated/decompensated)	—	25/25	53/47	10/10	0.99
	HBV-DNA (log10 IU/mL,mean)	—	3, 2	3, 2	3, 1	0.21
HC^c^	Number	15	50	100	8	
	Age (mean,SD)	43, 14	46, 16	43, 11	42, 14	0.37
	Gender (male/female)	10/5	35/15	72/28	13/7	0.91
	ALT (U/L,median,range)	—	19, 4–37	12, 9–39	10, 9–37	0.23

^a^CHB, chronic heptatis B. ^b^LC, liver cirrhosis.

^c^HC, healthy control.

^d^*p* value was calculated from, p < 0.05 was designated as significance.

**Table 2:** MiRNA profiles and diagnostic performance in training and validation datasets.

Targeted Disease vs Control	miRNA	Training Dataset			Validation Dataset		
		fold-change	*p*-value	AUC	fold-change	*p*-value	AUC
LC vs CHB	miR-18a-5p	0.5344	0.0047	0.664	0.4108	<0.0001	0.69
	miR-21-5p	0.711	0.0079	0.654	0.3962	<0.0001	0.76
	miR-29c-3p	0.3451	<0.0001	0.787	0.4564	<0.0001	0.767
	miR-106b-5p	0.509	0.0001	0.726	0.4567	<0.0001	0.686
	miR-122-5p	0.4433	0.0003	0.709	0.2796	<0.0001	0.787
	miR-185-5p	0.4472	0.0084	0.653	0.4555	<0.0001	0.703
LC vs HC	miR-1	3.5899	<0.0001	0.798	3.1534	<0.0001	0.879
	miR-146a-5p	3.6013	<0.0001	0.856	3.5424	<0.0001	0.919
	miR-451a	0.1242	<0.0001	0.894	0.1131	<0.0001	0.846
CHB vs HC	miR-21-5p	1.629	<0.0001	0.82	2.9823	<0.0001	0.927
	miR-27a-3p	3.3701	<0.0001	0.842	7.6229	<0.0001	0.937
	miR-122-5p	6.4798	<0.0001	0.853	10.0554	<0.0001	0.917
	miR-146a-5p	6.0375	<0.0001	0.946	10.7542	<0.0001	0.992

**Table 3:** MiRNA panels for the diagnosis of CHB and LC in validation datasets.

miRNA	Targeted Disease vs Control	Model	Cut-off value	Sensitivity	Specificity
Panel 1	LC vs CHB	Logit (P = LC) = 0.016 × miR-18a-5p − 0.022 × miR-21-5p + 0.013 × miR-29c-3p − 0.008 × miR-106b-5p − 0.007 × miR-122-5p − 0.002 × miR-185-5p +1.244	>0.6027	80%	70%
Panel 2	LC vs HC	Logit (P = LC) = 0.047 × miR-1 + 0.181 × miR-146a-5p − 0.006 × miR-451a − 4.076	>0.3041	95%	98%
Panel 3	CHB vs HC	Logit (P = CHB) = 0.027 × miR-21-5p − 0.079 × miR-27a-5p + 0.018 × miR-122-5p + 0.272 × miR-146a-5p − 7.562	>0.4325	97%	95%
